# From action intentions to action effects: how does the sense of agency come about?

**DOI:** 10.3389/fnhum.2014.00320

**Published:** 2014-05-15

**Authors:** Valérian Chambon, Nura Sidarus, Patrick Haggard

**Affiliations:** ^1^Laboratoire de Neurosciences Cognitives, INSERM U960Paris, France; ^2^Institut Jean Nicod, Ecole Normale Supérieure-EHESS, CNRS UMR-8129Paris, France; ^3^Institute of Cognitive Neuroscience, University College LondonLondon, UK

**Keywords:** fluency, action selection, agency, angular gyrus, human volition

## Abstract

Sense of agency refers to the feeling of controlling an external event through one’s own action. On one influential view, agency depends on how predictable the consequences of one’s action are, getting stronger as the match between predicted and actual effect of an action gets closer. Thus, sense of agency arises when external events that follow our action are consistent with predictions of action effects made by the motor system while we perform or simply intend to perform an action. According to this view, agency is inferred *retrospectively*, after an action has been performed and its consequences are known. In contrast, little is known about whether and how internal processes involved in the selection of actions may influence subjective sense of control, in advance of the action itself, and irrespective of effect predictability. In this article, we review several classes of behavioral and neuroimaging data suggesting that earlier processes, linked to fluency of action selection, *prospectively* contribute to sense of agency. These findings have important implications for better understanding human volition and abnormalities of action experience.

## Action-effect link and comparator models: a retrospective account of agency

Agency is a key component of action experience. In a nutshell, agency refers to the sense of controlling one’s own actions and, through these actions, events in the outside world. We rarely have an intense, clear phenomenology of agency, but we clearly recognize failures of agency when we experience actions that do not unfold as expected or fail to produce intended effects. One might even say that our sense of “authorship” becomes apparent only when it is falsified, resulting in a break of the flow from intentions to action effects that normally characterize experience. Thus, determining where the sense of agency comes from requires properly specifying where the break may occur along the intention-action-effect chain. Identifying the break may in turn depend on how we choose to specify the chain, and on the causal relation between its constituents (intention, action, effect).

**Figure 1:**
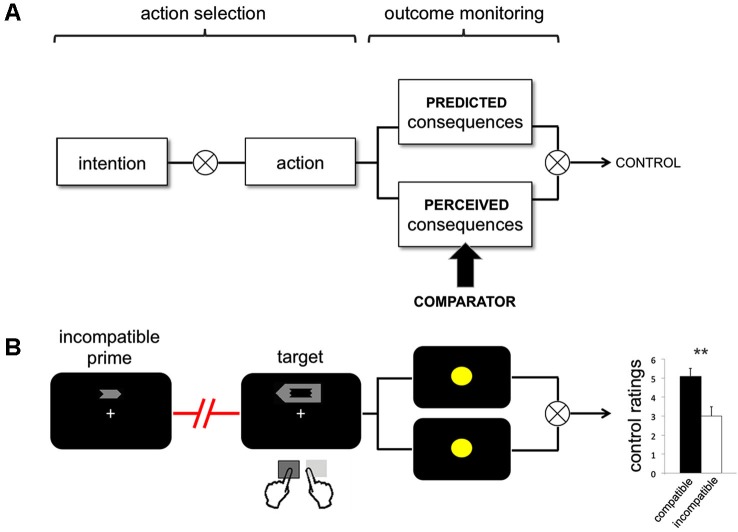
**(A)** Intention-Action-Effect chain. The action-selection processes operate between the formation of the initial intention and action execution. Dysfluency of action selection signals a break in the intention-action link, that occurs prior to the action and its sensory consequences. After the action has been selected, predicted and perceived consequences of this action are compared. On the comparator account (in bold), sense of agency is strong when there is a match between predicted and actually experienced consequences of an action, and is reduced in the case of a mismatch. **(B)** Example trial from the prime-target incompatible condition, adapted from Chambon et al. (2013). Participants were instructed to respond to the target stimulus, and were not informed of the presence of the arrow primes. Action effects consisted of colored circles that appeared on the screen after a varying delay. In this condition, sense of agency decreases relative to the compatible condition, even though predicted and perceived action effects are the same (yellow circles).

On one influential view, agency implies a control mechanism that causally relates actions to their effects. More specifically, it implies a mechanism that has goals, and that controls actions to achieve them. This mechanism was first, and successfully, formalized as a *comparator model* (Wolpert et al., 1995; Miall and Wolpert, 1996). In its first incarnation, a comparator model translates intentions into outcomes, by continually monitoring whether action consequences occur, or do not occur, as predicted. Though originally formulated as models of motor control (Wolpert et al., 1995), comparator models have also been increasingly used to explain the subjective sense of agency (e.g., Blakemore et al., 2001). On the comparator account, agency is computed by matching predicted and actually experienced consequences of movement. In this framework, action effects are precisely those sensory events that can be predicted from one’s intentions, using the specific intermediate mechanism of the comparator model (Wolpert et al., 1995; Figure 1A). Thus, the comparator model allows for two specific predictions. First, sense of agency should be strong when there is a close match between the predicted and the actual sensory consequences of an action, and should be reduced when predicted and experienced consequences do not match. Second, sense of agency necessarily occurs *late*, i.e., after an action has been performed, and sensory evidence about the consequences of action becomes available.

This view has received considerable empirical support from studies showing that spatial and temporal discrepancies between making an action and viewing visual feedback of the action reduce the sense that the observed action is one’s own. Thus, introducing a spatial transformation between an action and its visual consequences reduces participants’ sense of agency in proportion to the mismatch induced. In one typical task, participants received distorted visual feedback of their hand moving a joystick. When the movement of the virtual hand did not correspond to the subjects’ movement (Farrer and Frith, 2002), or when an angular bias was introduced between the subject’s and the virtual hand’s movement, participants more readily attributed it to another agent (Fourneret and Jeannerod, 1998; Farrer et al., 2003; Synofzik et al., 2006; David et al., 2007). Note that manipulating temporal relations between actions and outcomes had similar effects (Franck et al., 2001; Leube et al., 2003; MacDonald and Paus, 2003; David et al., 2007, 2011; Farrer et al., 2008). The so-called “intentional binding” effect provides another line of evidence for the role of temporal contiguity between action and outcome in the building of agency. The intentional binding effect has been first reported by Haggard et al. (2002): it refers to the subjective compression of the temporal interval between a voluntary action and its external sensory consequences. Thus, actions are perceived as shifted in time towards the outcomes that they cause, while outcomes are perceived as shifted back in time towards the actions that cause them (see Moore and Obhi, 2012, for a review). This temporal attraction is absent in cases of involuntary or passive movement. Equally, when participants simply judge the interval between action and effect, their judgments show a perceptual compression absent for equivalent passive movements (Engbert et al., 2008). The intentional binding effect would constitute an implicit, but reliable, measure of agency, as it only occurs when events in the external environment are precisely recognized as the *consequences* of one’s action.

On comparator accounts, a positive sense of agency is the default operation when no mismatch between predicted and current states occurs (see Synofzik et al., 2008). It is the experiential output of sub-personal processes that mostly run outside consciousness. Crucially, although sense of agency relies on real-time motor signals, it can only be computed after those signals are compared with reafferent feedback. Thus, a reliable, explicit sense of agency may only be formed when reafferent (visual, motor, or proprioceptive) signals become available for matching with intentions. Thus, one cannot feel agency over any event until that event has been registered and processed in the brain. As a consequence, agency can only be *retrospectively* attributed, although it is informed by *on-line* signals about motor guidance and control (Chambon and Haggard, 2013).

Note the retrospective account on agency has several advantages. First, it is grounded on several classes of converging behavioral and neuroimaging evidence. Second, it primarily relies on a computational model that provides a convincing explanation for the link between action and effect: action effects are sensory events that can be predicted from one’s action plans. However, an alternative possibility, that sense of agency is also generated *prospectively*, in advance of the action itself and before knowing the actual effect of actions, has received recent support (Wenke et al., 2010). On this view, selecting between alternative possible actions might itself generate a sense of agency. This view places a new emphasis on the intention-action, rather than the action-effect, link—i.e., on the process through which intentions are transformed into specific actions, to achieve desired effects. Importantly, this view suggests that agency may depend on real-time, prospective signals arising from internal circuits of action preparation, rather than on a *post-hoc*, retrospective comparison between predicted and current states of the environment.

## Intention-action link and selection fluency: a prospective account of agency

Previous studies have shown that judgments of agency tend to be related to how participants think that they perform in a task (Metcalfe and Greene, 2007). Similarly, errors in task performance may lead to a *feeling* of dysfluency during the task, without any explicit awareness of an error, and without the ability to explicitly report the error. Thus, a feeling that something went “wrong” during the control of instrumental action may be sufficient to modulate later judgments of control, even without being able to identify or explicitly report the error. The term “epistemic feeling” has been coined to describe this subjective, on-line, experience of an error (Arango-Muñoz, 2010; Charles et al., 2013). Importantly, such on-line experience strongly influences the sense of agency, as shown by recent priming studies. Thus, Wenke and colleagues showed that the sense of agency could be modulated by using subliminal priming to affect the *fluency* of action selection processes (Wenke et al., 2010; Haggard and Chambon, 2012, for a review). Interestingly, this procedure enabled a manipulation of the subjective sense of agency, without manipulating the* predictability* of action outcomes. In this experiment, participants pressed left or right keys in response to left- or right-pointing arrow targets. Prior to the target, subliminal left or right arrow primes were presented, unbeknownst to the subject. Prime arrow directions were either identical (compatible condition) or opposite (incompatible condition) to the subsequent target (Figure 1B). Responding to the target caused the appearance of a color after a jittered delay. The color patch can thus be considered as the action outcome. The specific color shown depended on whether the participant’s action was compatible or incompatible with the preceding subliminal prime, but did not depend on the prime identity or the chosen action alternative alone. Unlike previous studies, therefore, the primes did not predict action effects, nor could any specific color be predicted on the basis of the action chosen. Participants rated how much control they experienced over the different colors at the end of each block (Wenke et al., 2010).

Analyses of reaction times (RTs) showed that compatible primes facilitated responding whereas incompatible primes interfered with response selection. More importantly, priming also modulated the sense of agency over action effects: participants experienced more control over colors that followed actions compatible with the preceding primes than over colors that followed prime-incompatible actions. Thus subliminal priming made action selection processes more or less *fluent*, and this modulation of fluency affected the sense of agency over action outcomes.^1^

These results have several important cognitive implications. First, they suggest that the sense of agency depends strongly on processes of action selection that necessarily occur before action itself. Second, strong sense of agency may be associated with fluent, uncontested action selection. In contrast, conflict between alternative possible actions, such as that caused by incompatible subliminal priming, may reduce the feeling of control over action outcomes. Third, this prospective contribution of action selection processes to sense of agency is distinct from predicting the outcomes of action, since action outcomes were equally (un-) predictable for compatible and incompatible primes. That is, these primes did not prime effects of action as in previous studies (e.g., Wegner and Wheatley, 1999; Aarts et al., 2005; Linser and Goschke, 2007; Sato, 2009). Therefore, participants could not retrospectively base their control judgements on match between primes and effects alone. Rather, their stronger experience of control when primes were compatible could only be explained by the fluency of action selection—i.e., by a signal experienced *before* the action was made, and the effect was displayed.

Finally, participants did not consciously perceive the subliminal primes. Therefore, participants’ sense of agency could not be based on (conscious) beliefs about the primes. Instead, action priming itself presumably directly influenced the subjective sense of agency. Pacherie (Pacherie, 2008; see also Synofzik et al., 2008) has suggested that action selection conflict need not necessarily be conscious (Morsella et al., 2009). Such conflict may elicit the feeling “that something is wrong”, without necessarily leading to knowledge about* what* is wrong. Wenke et al.’s study shows that subjects can rely on this *implicit* *feeling* to make judgments about their own control over action effects^2^.

## Dissociating prospective sense of agency from motor performance

Wenke et al.’s findings suggest that monitoring fluency signals generated *during* action selection could be an important marker for the experience of agency. However, it is also possible that participants might have estimated agency based on implicit monitoring of their own performance, such as their RTs. Since RTs are lower on compatibly primed trials (Dehaene et al., 1998; Schlaghecken and Eimer, 2000; Schlaghecken et al., 2008), participants would therefore feel more control on compatible trials, because they respond more rapidly. On this second view, agency would depend on *retrospective* monitoring of action execution performance (Marti et al., 2010), not on *prospective* monitoring of premotor fluency signals.

To distinguish between these two accounts of sense of agency, we used an experimental procedure that dissociated fluency of action selection from performance monitoring (Chambon and Haggard, 2012). Specifically, we increased the interval between mask and target to take advantage of a Negative Compatibility Effect (NCE) in priming. Longer mask-target latencies *increase* RTs following compatible primes, relative to incompatible primes (Schlaghecken et al., 2008). By combining this factor with Wenke et al.’s design for assessing sense of agency, it was possible to directly compare the contrasting retrospective (performance monitoring) and prospective (action selection) accounts. Specifically, if sense of agency depends on selection fluency, it should be greater when actions are compatibly (fluent condition) versus incompatibly (dysfluent condition) primed, irrespective of whether priming benefits (faster RTs) or impairs (slower RTs) performance. Alternatively, if sense of agency depends only on performance monitoring, it should be stronger for rapid versus slower responding, irrespective of whether priming is compatible or incompatible with the action executed.

Crucially, reversing the normal relationship between prime-target compatibility and RTs did not alter subjective sense of agency. Thus, in compatible NCE trials, participants experienced *stronger* control despite *slower* response times and higher error rates, compared to incompatible NCE trials (Chambon and Haggard, 2012; see also Stenner et al., 2014). These results suggest that the feeling of control normally experienced by subjects on compatible trials does not depend on retrospectively monitoring performance, thereby strengthening the evidence for a prospective contribution of action selection fluency to sense of agency.

In both Wenke et al.’s (2010) and Chambon and Haggard’s (2012), experiments, priming did not influence the actual objective level of control that participants had over the colors presented after their actions. Indeed, the contingency between action and color effect was similar for compatibly-primed and incompatibly-primed trials. Importantly, the prospective sense of control identified in these experiments is therefore an illusion of control, since it is not based on differences in the actual statistical relation between action and effect. In other words, action selection is irrelevant to actual action-effect contingency, and thus to the agent’s actual ability to drive external events. Although illusory, this prospective sense of control may nevertheless be a convenient proxy for actual control, because agents often just know what to do and what will happen next in most everyday life situations. In that sense, *fluent* action selection is generally a good *advance predictor* of actual statistical control over the external environment (Haggard and Chambon, 2012; Chambon et al., 2014). Prospective agency might thus reflect a learned experiential metacognition: if we can fluently select an appropriate action, then we are likely to get what we want, or fulfill our intentions.

As suggested above, internal signals of premotor fluency might not produce a strong conscious experience with distinctive content, but might influence the experience of surrounding events. Thus, fluency of action selection would not be experienced as such, but would presumably be experienced as something that goes “right” or “wrong” in the control of instrumental action, and thus seems relevant to sense of agency. In that sense, signals relating to the fluency of action selection would not be perceived for what they really are, but (mis-)attributed to the processes of actually controlling the action. Such a misattribution may foster the subject not to adjust her behavior accordingly. Indeed, it has been shown that behavioral adjustment does not only depend on the presence or absence of an error, but also on its cause (e.g., me vs. not-me) (Steinhauser and Kiesel, 2011). Thus, if participants misattribute dysfluency to lack of control on the selected action, and misattribute fluency to the process of actually controlling the action, then they should adjust their behavior less in the dysfluent, than in the fluent, condition—despite the fact that control is equally illusory in both conditions. Future work is required to test this assumption directly.

## Neural substrates of prospective (fluency-based) agency

Taken together, these findings suggest that neural activity in action preparation circuits *prospectively* informs agency, independent of outcome predictability, and actual performance. Tracking dysfluency in action selection networks (Miele et al., 2011; Nahab et al., 2011) could be the basis for this prospective sense of agency. Recently, we adapted the prospective agency paradigm for functional neuroimaging (Chambon et al., 2013). Specifically, we studied whether the angular gyrus (AG), a parietal brain region which has been shown to compute *retrospective* agency by monitoring mismatches between actions and subsequent outcomes (Farrer et al., 2003, 2008), may also code for a *prospective* sense of agency, by monitoring action selection processes in advance of the action itself, and independently of action outcomes.

Behavioral results replicated those of Wenke and colleagues. Again, participants experienced greater control over action effects when the action was compatibly versus incompatibly primed (Chambon et al., 2013). More importantly, this prospective contribution of action-selection processes to sense of agency was accounted for by exchange of signals between specific frontal action selection areas and the parietal cortex. First, we found that activity in the AG was sensitive to mismatches, but not matches, between prime arrow and actual response to the target arrow. Moreover, this activity due to the prime-target mismatch predicted the magnitude of subsequent sense of agency: for incompatible trials only, activity in the AG decreased as sense of control over outcomes increased. Importantly, this neural coding of non-agency occurred at the time of action selection *only*, as in Wenke et al.’s original experiment.

**Figure 2:**
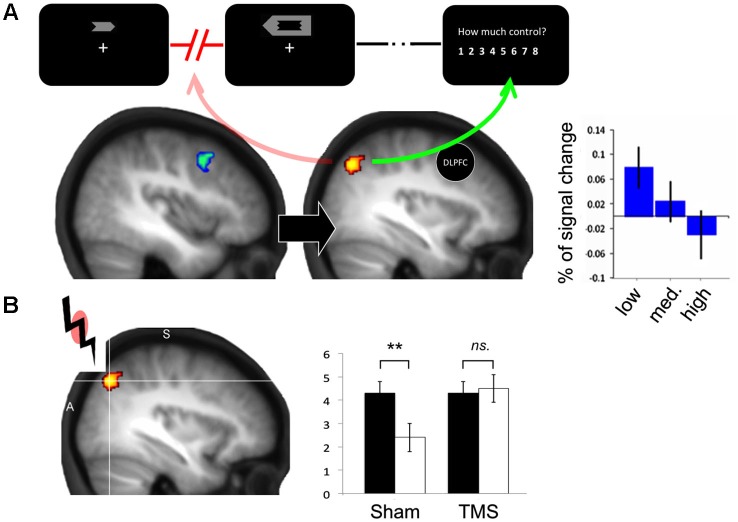
**(A)** Sense of agency is accounted for by exchange of signals across a prefrontal-parietal network: decreased DLPFC activity (in blue) due to incompatible primes results in a concomitant increase in AG activity (in yellow), and a subjective loss of control. The right chart shows a negative modulation of AG activity as a function of the level of experienced control (from low to high; adapted from Chambon et al., 2013). **(B)** TMS-induced disruption of left AG at the time of action selection abolishes the compatibility effect on sense of agency (right chart).

Second, connectivity analyses (psycho-physiological interaction) revealed that activity in the AG (signaling non-agency) in incompatible trials was negatively correlated with activity in the dorso-lateral prefrontal area (DLPFC; Figure 2A). Previous studies of willed action also noticed the same frontoparietal correlation, namely, that increased activity in DLPFC was associated with decreased activity in the AG (Frith et al., 1991). Our results are directly analogous: compatible primes might partly engage circuits for willed action, while prime-target incompatibility might relatively decrease activity in this circuit (Wenke et al., 2010). Thus, DLPFC *deactivation* would signal dysfluency in the selection of willed action, as a consequence of prime-target incompatibility. Decreased DLPFC activity due to incompatible primes would in turn result in a concomitant increase in AG activity and a subjective loss of control. Overall, this suggests that AG may monitor signals relating to fluency or dysfluency of action selection emanating from DLFPC and use them to (pre)construct an experience of agency. Importantly, under this interpretation, this monitoring of fluency signals by AG would occur *prior* to actions and their sensory consequences. This prospective contribution of AG to sense of agency can thus be distinguished from other functions such as action outcome monitoring. Interestingly, Farrer et al. (2008) demonstrated a role of AG in action outcome monitoring, but found a bilateral AG activation, which was slightly more ventral than the AG found here. In Farrer et al.’s study, AG activation varied with mismatch between predicted and actual sensory consequences of an action, while AG activation in Chambon et al.’s study was elicited by a mismatch between a prime-induced intention and response to a target (Chambon et al., 2013). The different localization found in these two studies could thus reflect a subdivision within the inferior parietal cortex, with more dorsal AG being involved in detecting mismatch between intention and action, independent of action consequences (Chambon et al., 2013), while more ventral AG would be involved in retrospectively comparing predicted and actual consequences of an action (Farrer et al., 2008).

Monitoring of fluency signals by AG might provide the subject with an on-line, subjective marker of volition, prior to action itself. As such, this finding sketches an important qualification of recent *post-hoc* determinist views of action control (Ackerman et al., 2010). In its strongest form, determinist views suggest that human behavior is unconsciously determined by subtle changes in the stimulus environment. On this view, individuals are not even aware of how their behavior is shaped and transformed, although they can retrospectively integrate general information about past actions and environmental cues to make inferences and narrative explanations about their own behavior (Wegner, 2002). While participants, in both Wenke’s and Chambon’s experiments, did not have any conscious experience of the subliminal primes, they did have a real-time *subjective experience* of their own action generation, which reflected the prime’s capacity to influence action selection. In this respect, the ability to monitor fluency signals generated *during* action selection in AG might be an important part of what makes our action intentional, and thus a key component of the experience of agency—defined as the feeling that we are intentionally making things happen by our own choices and actions.

## A causal evidence for the role of AG in the coding of prospective agency

Although informative, this fMRI study was nevertheless limited in two key ways. First, the evidence was indirect, because of the correlational nature of fMRI. Secondly, it was not possible to pinpoint the precise *time* at which AG is involved in the prospective coding of agency owing to the relatively poor temporal resolution of fMRI. As we saw, the issue of timing is important for understanding *where* the sense of agency is computed within the intention-action-effect chain.

We recently addressed these two limitations by combining single-pulse transcranial magnetic stimulation (TMS) with subliminal priming of action selection and judgements of control over action effects. On two distinct experiments we assessed the effects of TMS over left AG on action selection processing, by linking TMS to either (i) the presentation of the arrow target; (ii) to action execution; or (iii) to the presentation of the action effect (color patch). We made specific predictions based on our previous fMRI findings. Because AG activation correlated with sense of agency only on incompatible trials, we assumed that this area monitored signals relating to selection fluency generated by DLPFC (Chambon et al., 2013). In this case, applying TMS over AG should prevent this region from monitoring any signals from DLPFC, and hence reduce the tendency for incompatibility primes to influence judgements of control.

Consistent with these predictions, we found that TMS over left AG abolished the compatibility effect (i.e., the difference between compatible and incompatible conditions) on sense of agency at the time of action selection *only* (Figure 2B), while TMS delivered shortly after presentation of the action effect did not alter experienced agency. Importantly, TMS had no effect on RTs. This suggests that TMS-induced disruption of AG did not interfere with action selection processing itself, but rather interfered with a circuit that monitors selection fluency to pre-construct the experience of control.

Previously it is has been suggested that the AG is involved in the retrospective construction of sense of agency by monitoring the consistency between predicted and actual sensory consequences of movements (David et al., 2008, for a review). When these predictions are violated sense of agency is reduced, and AG activity is increased. Results from our TMS study do not disagree with this view of AG function, but point to an additional role: by monitoring the consistency between action plans and required actions, the AG is also involved in *earlier* prospective aspects of sense of agency, relating to action selection and action programming.

Note the prospective *and* retrospective mechanisms have some general features in common. Both involve monitoring action-related signals or “cues” (such as re-afferent sensory feedback) as they become available, and comparing them with other relevant information for consistency (see Moore et al., 2009). We suggest that monitoring and checking is a very general function of the AG during instrumental action. Initial action intentions, such as those caused by subliminal primes in the series of studies described above, could be checked for compatibility with the action subsequently performed. These action selection cues may provide an important “online” marker of control as the action is unfolding. Not only would this provide an estimate of agency without the need to wait until sensory feedback becomes available but, as we have suggested (Chambon et al., 2013), it may protect against aberrant experiences of agency. For example, the sense of agency in patients with schizophrenia is characterised by excessive reliance on re-afferent sensory information generated by their actions, presumably due to poor, or unreliable, action selection processing (Voss et al., 2010). Prospective signals—such as fluency signals—may indeed provide an important counterweight to re-afferent information, and hence may protect against xenopathic experiences (e.g., loss of control over one’s actions and thoughts) such as those experienced in passivity symptoms. At the same time, excessive reliance on these prospective signals may produce the opposite delusion of omnipotence, in which the mere decision to act is incorrectly assumed to produce successful action outcomes. This latter illusion appears to be common in historical despots but is interestingly absent in depressed people (Alloy and Abramson, 1979). A robust and reliable sense of agency may thus require a balanced—and probably context-dependent—mixture of both prospective and retrospective components. Future work is required to test whether other (contextual of individual) factors may influence the interplay between these two components. For example, it has been convincingly suggested that priming effects on the experience of agency depend on the *level* at which the agent represents her behavior (van der Weiden et al., 2010). Thus, while some people represent their own behavior at a low-level (i.e., the instrumental level: in terms of *how* an action is done), some others represent their behavior at a higher level (i.e., the outcome level: in terms of *why* an action is done). Interestingly, the former may depend more heavily on prospective cues to agency (e.g., selection fluency), whereas the latter may show excessive reliance on retrospective information—i.e., on general information about past actions and outcome-related cues.

## Linking fluency to outcome predictability

Recent accounts of agency have highlighted that it results from the integration of various cues (Synofzik et al., 2008; Moore and Fletcher, 2012), which may emerge at different times (Farrer et al., 2013). Namely, it has been suggested that several agency cues may be weighted by their reliability in order to obtain a “Bayesian optimal” estimate of true agency (Moore and Fletcher, 2012). This view has received some support as studies have shown, for example, that changes in action-contingency affected the weighting of predictive and postdictive cues (Moore and Haggard, 2008; Wolpe et al., 2013). As outcome predictability was reduced, there was a greater reliance on *post-hoc*, inferential processes.

In the action priming studies described above, outcomes were fully contingent on a given action, in order to hold outcome predictability constant. However, it remained unclear whether action selection fluency would still be a relevant cue to agency in a context of greater uncertainty about action-outcome relations. Given our previous proposal that action selection fluency could serve as an advance predictor of successful action (Chambon and Haggard, 2012), one might predict that reducing action-outcome contingency would reduce the contribution of the prospective (fluency-based) relative to the retrospective (outcome-based) cue, to sense of agency. That is, if the outcome monitoring revealed that the action was in fact unsuccessful—i.e., outcomes did not match expectations, then the fluency of action selection would no longer be relevant.

To test this, we adapted our previous paradigm (e.g., Chambon et al., 2013) to involve a reduced contingency between actions and outcomes (Sidarus et al., 2013). Thus, a given action was associated with two possible colored outcomes on 66% of trials, but these colors would appear after the alternative action on the remaining 33% of trials. This allowed us to create situations in which outcomes could either match or mismatch expectations, given action-outcome contingencies. In addition, these outcomes would follow actions that were either compatibly or incompatibly primed. Therefore, we could assess the relative contribution of a prospective cue—action selection fluency, with a retrospective cue—outcome monitoring.

Results showed that participants’ sense of agency was sensitive to manipulations of both the prospective and the retrospective cues. Compatibly primed actions were associated with higher control ratings than incompatibly primed actions. Additionally, participants reported a stronger sense of agency when the outcome was expected, compared to when the outcome was unexpected. More importantly, there was an interaction between the two variables. Incompatible action priming led to a significant reduction in control ratings when outcomes were unexpected, but not when outcomes were expected. At the same time, unexpected outcomes only reduced control ratings significantly when they followed incompatibly primed actions, and not compatibly primed actions (Sidarus et al., 2013). Thus, contrary to our predictions, selection fluency had a larger impact on sense of agency when outcomes were unexpected.

These findings reiterate the importance of action selection processes to the sense of agency. Even though outcomes were less predictable than in previous studies, we still found a similar effect of action priming on control ratings. What is more, the interaction between selection fluency and outcome expectation suggests that the sense of agency does not merely reflect information about action-outcome relations (e.g., Metcalfe and Greene, 2007). The sense of agency was drastically reduced only when both action selection was dysfluent *and* the outcome was unexpected. Prospective cues related to action selection fluency may thus make an independent contribution to the sense of agency from retrospective, outcome-based, cues.

Our findings are also not fully compatible with the cue integration models presently proposed for agency computation (Moore and Fletcher, 2012). Within this framework, it is the reliability of a given cue that determines its impact on the resulting sense of agency. Reliability is, however, a feature of the distribution of events. Thus, changes in cue reliability can only be assessed over a number of trials. Instead, our results suggest that the specific information carried by a given cue in a single trial can alter its weight relative to other cues. More complex Bayesian models of cue integration might be able to encompass these dynamic changes in cue weight. Yet, as mentioned above, perhaps a complete account of the sense of agency cannot be provided by simply maximising information about action-outcome relations.

These results overall support the idea that agency is the “default” assumption, which is only falsified, or reduced, when there is “sufficient” evidence against it.^3^ In some circumstances, it might be adaptive to maintain a sense of agency in the face of unexpected outcomes. Our environment mostly does not afford us fully predictable and contingent relations between actions and outcomes, but rather these tend to be probabilistic in nature. As such, we can learn these predictive relations, but we must also admit that predictions may be violated either due to the known statistical relations (e.g., when it is 66%), or due to random or outlier events. This type of *expected uncertainty* (Yu and Dayan, 2005) suggests that a mismatch between prediction and outcome does not always imply that the environment has changed, and one is not in control. In these situations, agency may be retained depending on information from other available cues, namely internal signals related to action selection.

## Action selection, agency, and expertise

Interestingly, the experience associated to selection fluency (at least partly) overlaps with the phenomenal properties of what has been formalized as “flow” in positive psychology (Csikszentmihalyi, 1990). The flow is a particular mental state, described by expert people as a feeling of mindfulness and total commitment to the task at hand, often associated with an experience of a dilation of subjective time (Witt and Sugovic, 2010; see also Hagura et al., 2012). In some professional tennis players, for example, this feeling of “flow”, resulting from a fluently selected (and perfectly executed) backhand stroke, may be associated with a “premonitory” anticipation of *where* the ball is going to hit the ground (Murphy and White, 1978). Consistently, our findings suggest that people may use the *fluency* (or ease) with which an action is selected as a good advance predictor of actual statistical control over the external environment.

Two hypotheses can be considered to account for the use of fluency signals in daily life. Using these signals adequately could first require learning *stable* relations between actions (e.g., the backhand stroke) and outcomes (e.g., where the tennis ball hit the court on average after that specific backhand). Indeed, simply having a feeling of fluently knowing which action to select does not guarantee the correct action outcome. Thus, fluency-based behaviors might only develop with expertise, once the brain has shifted from supervisory control to automatic or expert control. Under the expert regime, fluency would be used as an *implicit* *proxy* for the current status (success or failure) of the action unfolding (Haggard and Chambon, 2012), and would substitute for explicit monitoring of the action-effect link through short-circuiting the process of “checking” the actual consequences of our actions.

In contrast, an alternative hypothesis would propose that we learn in our everyday lives to use fluency of action selection as a reliable cue to agency. Fluency signals may become a heuristics for assessing one’s control over the external world, and we might even rely more on this heuristics in novel or uncertain situations. Before we know the statistical contingency between an action and its outcome in a given situation, we still have a sense of agency over what we do. Hence, we might rely on selection fluency to guide this sense of agency, until the more reliable action-outcome contingency cue is available. Although the Sidarus et al. (2013) study may provide some support for this alternative hypothesis, further research is needed to explore how the role of different agency cues may shift over time, during the learning of action-outcome relations. Similarly, high levels of expertise in complex tasks may involve the recruitment of different processes, and also affect the types of cues that inform the sense of agency.

## Conflict of interest statement

The authors declare that the research was conducted in the absence of any commercial or financial relationships that could be construed as a potential conflict of interest.

## Footnotes

^1^Subliminal priming would facilitate action selection by reducing conflict between alternative action programmes (Fleming et al., 2009). This facilitation of premotor processing would precisely be experienced as a feeling of action fluency, while the opposite effect of conflict between alternative actions would be experienced as dysfluency. Note this suggestion is analogous to the wellaccepted way that the feeling of “effort” is seen, as the experiential output of an increased demand in cognitive control (McGuire and Botvinick, 2010). Conversely, the literature usually defines fluency as an experiential consequence of smooth, effortless cognitive processing (e.g., Oppenheimer, 2008). In this sense, both terms are interchangeable—a fluent processing is an effortless processing–, and both may prospectively inform agency (e.g., Demanet et al., 2013).

^2^We take that selection fluency does not require to be explicitly represented to informconscious experience of action. In fact, recent data suggest that fluency signals need to be kept implicit in order to influence agency on compatible trials–i.e., in order to be mistaken for actual control over action effects. Indeed, when primes are consciously perceived (i.e., presented at a supraliminal threshold), the compatibility effect is reversed: sense of agency is higher on incompatible trials (Damen et al., 2014).

^3^Whereas subjects experience less control when presented with incompatibly vs. neutrally-primed (using “neutral” primes constructed by superimposing left- and right-oriented primes), they do not feel more control when compatibly vs. neutrally-primed (Chambon and Haggard, 2012). These results are consistent with previous accounts suggesting that agency is a default experience. On this view, sense of agency would only really become apparent as a sense of non-agency, when the normal flow from our intentions to the effects of our actions is broken (Haggard, 2005; Chambon and Haggard, 2013).
